# Supplementary material for: In Situ Conservation of Orchidaceae Diversity in the Intercontinental Biosphere Reserve of the Mediterranean (Moroccan Part)
Source: Plants (Basel). 2025 Apr 20;14(8):1254. doi: 10.3390/plants14081254 (PMC12030076; doi:10.3390/plants14081254)
Supplement: Supplementary file 1 [file plants-14-01254-s001.zip › Suplementary materiels Figure S1 and table S1 and Appendix S1/_Appendix S1.pdf]

Article

In Situ Conservation of Orchidaceae Diversity in the Intercontinental Biosphere Reserve of the Mediterranean (Moroccan Part)

Yahya El Karmoudi [1], Nikos Krigas [2,3,*], Brahim Chergui El Hemiani [1], Abdelmajid Khabbach [4] and Mohamed Libiad [1,*]

[1] Ecology, Systematics and Biodiversity Conservation Laboratory, URL-CNRST N° 18, FS, Abdelmalek Essaadi University, M'Hannech II, Tetouan 93002, Morocco; yahyaelkarmoudi@gmail.com (Y.E.K.); b.cherguielhemiani@uae.ac.ma (B.C.E.H.)

[2] Institute of Plant Breeding and Genetic Resources, Hellenic Agricultural Organisation Demeter (ELGO-DIMITRA), 57001 Thessaloniki, Greece

[3] Department of Viticulture, Floriculture & Plant Protection, Institute of Olive Tree, Subtropical Crops and Viticulture, Hellenic Agricultural Organization Demeter (ELGO-DIMITRA), 71307 Heraklion, Greece

[4] Biotechnology, Environment, Agri-Food and Health Laboratory, Faculty of Sciences Dhar El Mahraz, Sidi Mohamed Ben Abdellah University, Fès 30003, Morocco; khamajid@hotmail.com (A.K.)

* Correspondence: nkrigas@elgo.gr (N.K.); libiad001@gmail.com (M.L.)

Supplementary material

Appendix S1. Floristic catalogue and distribution maps of the recorded orchid taxa in the study area

The following floristic catalogue presents the recorded orchid taxa in the study area (nomenclature of taxa according to POWO [37], referred in brackets when different according to APD [36], and their distribution in the study area (Figure 1), annotating also the presence of each taxon in the areas and the specific sites surveyed per altitude, substrate, and habitats (see Table 1), along with the recorded number of orchid individuals (numbers in parentheses) and comments regarding new floristic reports per region compared to previous studies [11-13], with different information fields separated by column (;) while asterisks (*) indicate unresolved taxonomic issues:

***Cephalanthera longifolia* (L.) Fritsch**

Talassemtane National Park; 5(1), 6(9), 13(5), 18(23),19(12), 24(4); 415-1366 m; calcareous substrate; matorral and rainforest with *Q. ilex* and matorral with *Pistacia lentiscus* L. and *Chamaerops humilis* L.

***Epipactis tremolsii* Pau**

Talassemtane National Park; 18(18), 19(10), 20(8); 1318-1573 m; calcareous substrate; rainforest with *Q. ilex*

***Himantoglossum hircinum* (L.) Spreng.**

Talassemtane National Park; 8(2), 12(3); 1174-1608 m; calcareous substrate; rainforest with *Abies pinsapo* subsp. *marocana* and *Q. ilex*

***Himantoglossum robertianum* (Loisel.) P.Delforge**

Talassemtane National Park; 3(2); 1147 m; calcareous substrate; lawns with Asteraceae, Boraginaceae and Poaceae; new record in the area

Dardara; 33(14), 34 (120), 35 (30); 306-349m; calcareous substrate; rainforest with *Q. suber, P. lentiscus, Myrtus communis* L.; new record in the area

***Limodorum trabutianum* Batt.**

Talassemtane National Park; 19(3); 1318 m; calcareous substrate; rainforest with *Quercus faginea* and *Q. ilex*

Bouhachem Natural Park; 31(4), 32(1); 1471-1522 m; rainforest on siliceous substrate with *Quercus canariensis, Q. lusitanica, Q. pyrenaica*

***Neotinea maculata* (Desf.) Stearn [*Orchis intacta* Link]**

Talassemtane National Park; 6(12); 11(16), 12(4), 13(8), 14(10); 1156- 1715 m; calcareous substrate; coniferous forest with *A. pinsapo* subsp. *marocana* and matorral with *Q. ilex*

Dardara; 37(13); 330 m; calcareous substrate; rainforest with *Q. suber*

***Ophrys apifera* Huds.**

Dardara; 37(26); 330 m; calcareous substrate; rainforest with *Q. suber*

J. Lahbib; 38(23), 39(15), 40(21); 77-88m; calcareous substrate; matorral with *Pistacia atlantica* Desf., *Olea europaea* L. subsp. *europaea*, *Ch. humilis* and matorral with *P. lentiscus, M. communis;* new record in the area

*** *Ophrys fusca* Link subsp. *fusca* [*Ophrys flammeola* P.Delforge]**

Talassemtane National Park; 12(4), 14(1), 22(5); 1608-1664 m; calcareous substrate; coniferous forest with *A. pinsapo* subsp. *marocana, Juniperus oxycedrus* L., *Berberis vulgaris* subsp. *australis* (Boiss.) Heywood

****Ophrys lutea* Cav. subsp. *galilaea* (H.Fleischm. & Bornm.) Soó [*Ophrys sicula* Tineo]**

Talassemtane National Park; 12(1), 14(8), 20(12); 1608-1664 m; calcareous substrate; coniferous forest of *A. pinsapo* subsp. *marocana, J. oxycedrus* and *B. vulgaris* subsp. *australis* and rainforest with *Q. ilex* and *J. oxycedrus*

****Ophrys* x *battandieri* E.G.Camus [*Ophrys battandieri* E.G.Camus, *Ophrys numida* Devillers-Tersch. & Devillers]**

J. Lahbib; 41(9); 107 m; calcareous substrate; matorral with *P. lentiscus,* and *M. communis;* new record in the area

Talassemtane National Park; 12(6), 13(8), 14(9), 22(21), 23(16); 1313-1664 m; calcareous substrate; coniferous forest with *A. pinsapo* subsp. *marocana* and matorral with *Q. ilex*

***Ophrys scolopax* Cav. subsp. *apiformis* (Desf.) Maire & Weiller**

Talassemtane National Park; 16(1); 1026 m; calcareous substrate; chamaephytic steppe with *Ch. humilis, Stachys fontqueri* Pau and *Cistus albidus* L.

J. Lahbib; 41(8); 107 m; calcareous substrate; matorral with *P. lentiscus, M. communis;* new record in the area

***Ophrys speculum* Link**

J. Lahbib; 41(3), 42(29); 104-107m; calcareous substrate; matorral with *P. lentiscus, M. communis;* new record in the area

**** Ophrys tenthredinifera* Willd. [*Ophrys tenthredinifera* Willd. subsp. *grandiflora* (Ten.) Kreutz, *Ophrys tenthredinifera* Willd. subsp. *ficalhoana* (J.A.Guim.) M.R.Lowe & D.Tyteca, *Ophrys tenthredinifera* Willd. subsp. *tenthredinifera*]**

Talassemtane National Park; 1(3), 6(4), 10(42), 13(19), 15(4), 19(36), 20(14), 21(8); 1156-1876 m; calcareous substrate; rainforest and matorral with *Q. ilex*

**** Ophrys tenthredinifera* Willd. [*Ophrys tenthredinifera* Willd. subsp. *Tenthredinifera]***

J. Lahbib; 41(21); 107 m; calcareous substrate; matorral with *P. lentiscus* and *M. communis;* new record in the area

***Orchis anthropophora* (L.) All.**

Talassemtane National Park; 11(8), 12(40), 23(18); 1352-1715 m; calcareous substrate; coniferous forest with *A. pinsapo* subsp. *marocana*

***Orchis mascula* (L.) L. [*Androrchis mascula* (L.) D.Tyteca & E.Klein]**

Talassemtane National Park; 4(12), 17(2), 20(3); 1112-1573 m; calcareous substrate; matorral and rainforest with *Q. ilex;* new record in the area

Bouhachem Natural Park; 28(10); 1113 m; siliceous substrate; rainforest based on *Q. suber* and *Q. pyrenaica;* new record in the area

***Orchis mascula* (L.) L. subsp. *laxifloriformis* Rivas Goday & B.Rodr. *[Androrchis langei* (K.Richt.) D.Tyteca & E. Klein]**

Talassemtane National Park; 2(20), 7(34), 8(12), 9(15), 13(23); 1149-1969 m; calcareous substrate; matorral and rainforest with *Quercus ilex*

Bouhachem Natural Park; 26(12), 27(6), 31(14), 32(20); 849-1522 m; siliceous substrate; Natural rainforest based on *Quercus canariensis* Willd., *Quercus lusitanica* Lam., *Quercus pyrenaica* Willd. and degraded rainforest with *Quercus suber* L. and *Pteridium aquilinum* (L.) Kuhn

***Orchis spitzelii* Saut. ex W.D.J.Koch subsp. *cazorlensis* (Lacaita) D.Rivera & Lopez Velez *[Orchis cazorlensis* Lacaita]**

Talassemtane National Park; 11(13); 1715 m; calcareous substrate; coniferous forest with *A. pinsapo* subsp. *marocana;* new record in the area

Serapias lingua* L. subsp. *lingua

Talassemtane National Park; 7(17); 1174 m; calcareous substrate; rainforest with *Q. ilex;* new record in the area

Dardara; 36(16); 373 m; calcareous substrate; rainforest with *Q. suber;* new record in the area

Bouhachem Natural Park; 29(14), 30(13); 287-712 m; siliceous substrate; rainforest with *Q. suber* and *Q. pyrenaica*

***Serapias parviflora* Parl.**

Talassemtane National Park; 7(30), 24(3); 415-1174 m; calcareous substrate; rainforest with *Q. ilex* and matorral with *P. lentiscus* and *Ch. humilis*

Dardara; 37(18); 330 m; calcareous substrate; rainforest based on with *Q. suber*

J. Lahbib; 39(8), 41(6); 77-107 m; calcareous substrate; matorral with *P. lentiscus*, *M. communis;* new record in the area

Bouhachem Natural Park; 25(11); 1293 m; siliceous substrate; rainforest with *Q. suber* and *Q. pyrenaica*

***Serapias strictiflora* Welw. ex Veiga**

Dardara; 36(8); 373 m; calcareous substrate; rainforest with *Q. suber;* new record in the area

Bouhachem Natural Park; 30(11); 287 m; siliceous substrate; rainforest with *Q. suber, Q. pyrenaica*

***Serapias vomeracea* (Burm.f.) Briq.**

Bouhachem Natural Park; 29(12); 712 m; siliceous substrate; rainforest with *Q. suber, Q. pyrenaica;* new record in the area
