# Supplementary material for: In Situ Conservation of Orchidaceae Diversity in the Intercontinental Biosphere Reserve of the Mediterranean (Moroccan Part)
Source: Plants (Basel). 2025 Apr 20;14(8):1254. doi: 10.3390/plants14081254 (PMC12030076; doi:10.3390/plants14081254)
Supplement: Supplementary file 1 [file plants-14-01254-s001.zip › Suplementary materiels Figure S1 and table S1 and Appendix S1/_Supplementary materials Figure S1 and Table S1.pdf]

Article

In Situ Conservation of Orchidaceae Diversity in the Intercontinental Biosphere Reserve of the Mediterranean (Moroccan Part)

Yahya El Karmoudi [1], Nikos Krigas [2,3,*], Brahim Chergui El Hemiani [1], Abdelmajid Khabbach [4] and Mohamed Libiad [1,*]

[1] Ecology, Systematics and Biodiversity Conservation Laboratory, URL-CNRST N° 18, FS, Abdelmalek Essaadi University, M'Hannech II, Tetouan 93002, Morocco; yahyaelkarmoudi@gmail.com (Y.E.K.); b.cherguielhemiani@uae.ac.ma (B.C.E.H.)

[2] Institute of Plant Breeding and Genetic Resources, Hellenic Agricultural Organisation Demeter (ELGO-DIMITRA), 57001 Thessaloniki, Greece

[3] Department of Viticulture, Floriculture & Plant Protection, Institute of Olive Tree, Subtropical Crops and Viticulture, Hellenic Agricultural Organization Demeter (ELGO-DIMITRA), 71307 Heraklion, Greece

[4] Biotechnology, Environment, Agri-Food and Health Laboratory, Faculty of Sciences Dhar El Mahraz, Sidi Mohamed Ben Abdellah University, Fès 30003, Morocco; khamajid@hotmail.com (A.K.)

* Correspondence: nkrigas@elgo.gr (N.K.); libiad001@gmail.com (M.L.)

Supplementary materials

Figure S1. The distinct occurrences of the three subspecies of *Ophrys tenthredinifera* Willd. according to APD [36]

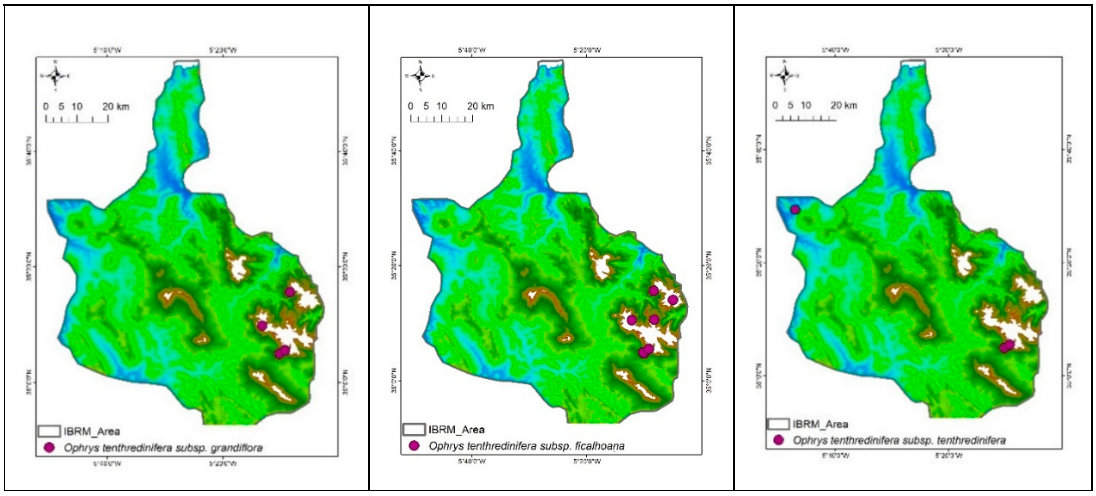

Table S1. Catalogue of non-Orchidaceae vascular plants recorded in the Moroccan part of the Intercontinental Biosphere Reserve of the Mediterranean

Families	Species or subspecies	Areas	Sites
		Dominant species	
Anacardiaceae	*Pistacia atlantica* Desf.	Jbel Lahbib	38
Anacardiaceae	*Pistacia lentiscus* L.	Dardara	33, 34
		Jbel Lahbib	39, 40, 41
		Talassemtane National Park	24
Arecaceae	*Chamaerops humilis* L.	Talassemtane National Park	16, 24
		Jbel Lahbib	38
Dennstaedtiaceae	*Pteridium aquilinum* (L.) Kuhn	Bouhachem Natural Park	26, 27
Fagaceae	*Quercus faginea* Lam.	Talassemtane National Park	19
	Quercus canariensis Willd.	Bouhachem Natural Park	31, 32
	Quercus ilex L.	Talassemtane National Park	1, 2, 5, 6, 7, 8, 9, 10, 13, 15, 17, 18, 19, 20, 21
	Quercus lusitanica Lam.	Bouhachem Natural Park	31, 32
	Quercus pyrenaica Willd.	Bouhachem Natural Park	25, 28, 29 30, 31, 32
	Quercus suber L.	Bouhachem Natural Park	25, 28, 29, 30
		Dardara	33, 34, 35, 36, 37
Lamiaceae	*Stachys fontqueri* Pau	Talassemtane National Park	16
Myrtaceae	*Myrtus communis* L.	Dardara	33, 34, 35, 36, 37
		Jbel Lahbib	39, 40, 41
Oleaceae	*Olea europaea* L. subsp. *europaea*	Jbel Lahbib	38
Pinaceae	*Abies pinsapo* subsp. *marocana* (Trab.) Emb. & Maire [*Abies marocana* Trab.]	Talassemtane National Park	11, 12, 14, 22, 23
		Companion species	
Adoxaceae	*Viburnum tinus* L.	Talassemtane National Park	18
Amaryllidaceae	*Narcissus cantabricus* DC.	Talassemtane National Park	19
Anacardiaceae	*Pistacia lentiscus* L.	Dardara	35, 36, 37
		Talassemtane National Park	19, 20, 21
Araceae	*Arisarum vulgare* O.Targ.Tozz.	Dardara	33, 34
		Jbel Lahbib	40, 41
Asparagaceae	*Drimia maritima* (L.) Stearn	Bouhachem Natural Park	26
Asteraceae	*Scolymus hispanicus* L.	Jbel Lahbib	42
	Cynara sp.	Talassemtane National Park	12, 14

	Dittrichia viscosa (L.) Greuter		16
	Eryngium sp.		10, 11, 13
Berberidaceae	*Berberis vulgaris* subsp. *australis* (Boiss.) Heywood [*Berberis hispanica* Boiss. & Reut.]	Talassemtane National Park	12, 22, 23
Boraginaceae	*Lithodora* sp.	Talassemtane National Park	18
Cistaceae	*Cistus albidus* L.	Bouhachem Natural Park	27
		Jbel Lahbib	40, 41
		Talassemtane National Park	9, 10, 11, 13, 16, 17, 18, 18, 19, 20, 21
	Cistus crispus L.	Bouhachem Natural Park	25, 26, 27, 31, 32
		Jbel Lahbib	39
		Talassemtane National Park	6, 7, 10, 13,
	Cistus ladanifer L.	Talassemtane National Park	6, 7, 10, 13
	Cistus monspeliensis L.	Dardara	33, 34, 36, 37
		Jbel Lahbib	38, 39, 40, 41
		Talassemtane National Park	8, 10
	Cistus salviifolius L.	Bouhachem Natural Park	28, 29
		Talassemtane National Park	5, 17
Cupressaceae	*Juniperus oxycedrus* L.	Bouhachem Natural Park	31, 32
		Dardara	33, 34, 36, 37
		Jbel Lahbib	42
		Talassemtane National Park	11, 12, 20, 21, 22
Cyperaceae	*Cyperus* sp.	Talassemtane National Park	10
Dennstaedtiaceae	*Pteridium aquilinum* (L.) Kuhn	Bouhachem Natural Park	28, 29
Ericaceae	*Arbutus unedo* L.	Bouhachem Natural Park	28, 29, 30, 31, 32
		Dardara	36, 37
		Talassemtane National Park	6, 7, 10, 13, 17, 18
	Erica arborea L.	Jbel Lahbib	39, 40, 41
		Bouhachem Natural Park	28, 29
		Dardara	36, 37
Euphorbiaceae	*Euphorbia* sp.	Talassemtane National Park	10, 17
Fabaceae	*Adenocarpus* sp.	Talassemtane National Park	18
	Calicotome villosa (Poir.) Link	Dardara	34, 35, 36, 37
		Jbel Lahbib	38
	Cytisus sp.	Bouhachem Natural Park	30
	Erinacea anthyllis Link.	Talassemtane National Park	12
Fagaceae	*Quercus ilex* L.	Talassemtane National Park	22
Iridaceae	*Romulea* sp.	Bouhachem Natural Park	25
	Iris sp.	Talassemtane National Park	16
Lamiaceae	*Mentha* sp.	Bouhachem Natural Park	26, 28, 29

	Origanum elongatum (Bonnet) Emb. & Maire	Talassemtane National Park	10, 13, 16
	Teucrium embergeri (Sauvage & Vindt) El Oualidi, T.Navarro & al.	Talassemtane National Park	16, 22
Asphodelaceae	*Asphodelus* sp.	Bouhachem Natural Park	25, 26, 27, 31, 32
		Jbel Lahbib	38
Oleaceae	*Olea europaea* L. subsp. *europaea*	Dardara	33, 34
Orobanchaceae	*Orobanche* sp.	Bouhachem Natural Park	28
Paeoniaceae	*Paeonia coriacea* Boiss.	Talassemtane National Park	18
Pinaceae	*Pinus halepensis* Mill.	Talassemtane National Park	22, 23, 24
	Pinus nigra J.F.Arnold subsp. *salzmannii* (Dunal) Franco	Talassemtane National Park	11
Plantaginaceae	*Plantago* sp.	Jbel Lahbib	42
		Talassemtane National Park	10
Ranunculaceae	*Ranunculus* sp.	Bouhachem Natural Park	28, 29, 30
Rosaceae	*Crataegus monogyna* Jacq.	Dardara	36, 37
		Talassemtane National Park	11, 23
	Rubus ulmifolius Schott	Bouhachem Natural Park	30
		Talassemtane National Park	9, 22
Thymelaeaceae	*Daphne gnidium* L.	Dardara	36, 37
		Talassemtane National Park	10, 13, 18, 19
	Daphne laureola L.	Talassemtane National Park	8, 22
Violaceae	*Viola* sp.	Talassemtane National Park	23
